# Antithrombin III Levels and Outcomes Among Patients With Trauma

**DOI:** 10.1001/jamanetworkopen.2024.27786

**Published:** 2024-08-15

**Authors:** David H. Farrell, Keeley M. McConnell, Jevgenia Zilberman-Rudenko, Brandon Behrens, Scott Mcloud, Mackenzie R. Cook, David Martin, John D. Yonge, Samantha J. Underwood, Diane E. Lape, Andrew Goodman, Martin A. Schreiber

**Affiliations:** 1 Donald D. Trunkey Center for Civilian and Combat Casualty Care, Oregon Health & Science University, Portland; 2 Division of Acute Care Surgery, Department of Surgery, University of Minnesota, Minneapolis

## Abstract

**Importance:**

Patients with trauma exhibit a complex balance of coagulopathy manifested by both bleeding and thrombosis. Antithrombin III is a plasma protein that functions as an important regulator of coagulation. Previous studies have found a high incidence of antithrombin III deficiency among patients with trauma.

**Objective:**

To assess whether changes in antithrombin III activity are associated with thrombohemorrhagic complications among patients with trauma.

**Design, Setting, and Participants:**

This cohort study was conducted from December 2, 2015, to March 24, 2017, at a level I trauma center. A total of 292 patients with trauma were followed up from their arrival through 6 days from admission. Data, including quantification of antithrombin III activity, were collected for these patients. Thromboprophylaxis strategy; hemorrhage, deep vein thrombosis (DVT), and pulmonary embolism screenings; and follow-up evaluations were conducted per institutional protocols. Data analyses were performed from September 28, 2023, to June 4, 2024.

**Main Outcomes and Measures:**

The primary study outcome measurements were associations between antithrombin III levels and outcomes among patients with trauma, including ventilator-free days, hospital-free days, intensive care unit (ICU)–free days, hemorrhage, venous thromboembolic events, and mortality.

**Results:**

The 292 patients had a mean (SD) age of 54.4 (19.0) years and included 211 men (72.2%). Patients with an antithrombin III deficiency had fewer mean (SD) ventilator-free days (27.8 [5.1] vs 29.6 [1.4]; *P* = .0003), hospital-free days (20.3 [8.2] vs 24.0 [5.7]; *P* = 1.37 × 10^−6^), and ICU-free days (25.7 [4.9] vs 27.7 [2.3]; *P* = 9.38 × 10^−6^) compared with patients without a deficiency. Antithrombin III deficiency was also associated with greater rates of progressive intracranial hemorrhage (21.1% [28 of 133] vs 6.3% [10 of 159]; *P* = .0003) and thrombocytopenia (24.8% [33 of 133] vs 5.0% [8 of 159]; *P* = 1.94 × 10^−6^). Although antithrombin III deficiency was not significantly associated with DVT, patients who developed a DVT had a more precipitous decrease in antithrombin III levels that were significantly lower than patients who did not develop a DVT.

**Conclusions and Relevance:**

In this cohort study of patients with trauma, antithrombin III deficiency was associated with greater injury severity, increased hemorrhage, and increased mortality, as well as fewer ventilator-free, hospital-free, and ICU-free days. Although this was an associative study, these data suggest that antithrombin III levels may be useful in the risk assessment of patients with trauma.

## Key Points

**Question:**

Is antithrombin III deficiency associated with thrombohemorrhagic complications among patients with trauma?

**Findings:**

In this cohort study of 292 patients with trauma, antithrombin III deficiency was associated with fewer ventilator-free days, hospital-free days, intensive care unit–free days and lower platelet counts, as well as greater rates of progressive intracranial hemorrhage. Patients who experienced venous thromboembolic events had decreased antithrombin III levels after admission.

**Meaning:**

This study suggests that antithrombin III levels may be useful in the risk assessment of patients with trauma.

## Teaser

This cohort study assesses whether antithrombin III deficiency is associated with thrombohemorrhagic complications among patients with trauma.

## Introduction

Patients with trauma exhibit a complex balance of coagulopathy that is only partially understood.^1,2,3^ Despite thromboprophylaxis suggested by the American College of Chest Physicians,^4^ rates of deep vein thrombosis (DVT) remain high in this cohort.^5^ The threat to life is highlighted by the finding that pulmonary embolism (PE) is the third most common cause of death for trauma patients who survive the first 24 hours of hospitalization.^6,7,8^ Thromboprophylaxis strategies weigh risks of venous thromboembolic events (VTEs) and hemorrhage, a consideration that becomes especially complex in the more severely ill and frail population of older adults.^9^

Antithrombin III is a natural anticoagulant with additional roles pertaining to vascular barrier function.^10,11^ Antithrombin III levels change after major surgery or injury and are affected by a number of physiologic processes as well as standard resuscitative efforts in trauma centers. Trauma patients commonly have antithrombin III deficiency, which has been hypothesized to predispose this cohort to greater rates of VTE^12,13^ and, more recently, suggested to play a role in thrombohemorrhagic complications among trauma patients.^14^ In this study, we examined the changes in antithrombin III levels among our trauma cohort and the association of these changes with certain outcomes. We hypothesized that antithrombin III deficiency after injury would be associated with hemorrhagic complications, VTEs, and mortality. If this hypothesis is confirmed, antithrombin III could be a useful and easily measured biomarker to help stratify risk profiles of hospitalized trauma patients.

## Methods

### Study Design

This was a prospective, observational, single-institution cohort study performed from December 2, 2015, to March 24, 2017, at Oregon Health & Science University (OHSU), a level I trauma center. This study was approved by the OHSU institutional review board and was carried out in accordance with The Code of Ethics of the World Medical Association (Declaration of Helsinki).^15^ Participant consent was obtained using an institutional review board–approved consent form. Study data were collected and managed using Research Electronic Data Capture (REDCap) tools hosted at OHSU.^16,17^ REDCap is a secure, web-based software platform designed to support data capture for research studies, providing (1) an intuitive interface for validated data capture, (2) audit trails for tracking data manipulation and export procedures, (3) automated export procedures for seamless data downloads to common statistical packages, and (4) procedures for data integration and interoperability with external sources. This study followed the Strengthening the Reporting of Observational Studies in Epidemiology (STROBE) reporting guideline.

**Figure 1.**
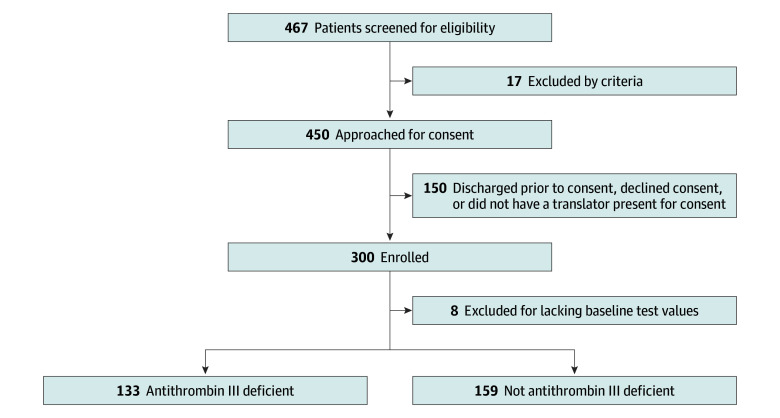
Study Flow Diagram

**Figure 2.**
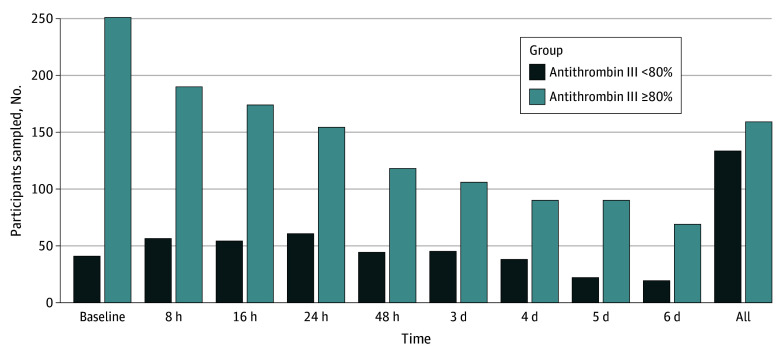
Sampling of Antithrombin III–Deficient Patients Across Time Data represent the breakdown of patients with antithrombin III deficiency during the study.

After appropriate screening (Figure 1; eFigure in Supplement 1), laboratory data were collected on 292 trauma patients at the following time points: admission (baseline), 8 hours, 16 hours, 24 hours, 48 hours, and days 3, 4, 5, and 6 after the initial sample collection (Figure 2). Not all patients were sampled at every time point. For the thromboprophylaxis strategy, patients were placed on the institutional protocol regimen of 30 mg of enoxaparin twice a day as soon as no contraindications were present. Any decisions to withhold doses for clinical changes or procedures were made by the primary clinical team. All patients underwent protocolized screening for complete leg lower extremity DVT with duplex ultrasonography performed on hospital day 3, hospital day 7, and weekly thereafter until hospital discharge or death. The decision to evaluate for PE and/or hemorrhage was made by the primary treatment team based on the patient’s clinical status. If a diagnosis of DVT or PE was made based on results of imaging, patients were treated with therapeutic anticoagulation with enoxaparin or a continuous intravenous infusion of heparin as per institutional protocol. Patients were followed up to hospital discharge to assess for DVT or PE, and patients with diagnosed DVT or PE were discharged with a therapeutic oral anticoagulation regimen. Hemorrhage was defined as an episode of systolic blood pressure less than 90 mm Hg accompanied by the need for a blood transfusion or evidence of vascular extravasation on pertinent imaging. Progression of intracranial hemorrhage (ICH) was tracked on repeat computed tomography of the head as interpreted by the attending radiologist, who was blinded to antithrombin III levels.

### Inclusion and Exclusion Criteria

Any trauma activation patient meeting physiological or anatomical criteria indicating high risk for life-threatening injuries was enrolled; full inclusion and exclusion criteria are summarized in the eFigure in Supplement 1. Patients younger than 18 years and transferred patients who underwent an invasive procedure or received blood products prior to arrival at OHSU or who failed to have blood samples drawn within 1 hour of admission were excluded. In addition, prisoners and patients enrolled in a prehospital intervention study were excluded.

### Laboratory Investigations

Samples run from each time point included complete blood count, activated partial thromboplastin time, anti-factor Xa level, prothrombin time, international normalized ratio, and antithrombin III activity level. Antithrombin III activity was measured via a chromogenic functional assay (Diagnostica Stago Inc) and reported as a percentage of the standard control, with a reference range of normal being 80% to 120% activity.

### Statistical Analysis

Statistical analyses were performed from September 28, 2023, to June 4, 2024, after the close of the study using RStudio, version 2023.06.1 Build 524 (R Project for Statistical Computing). For reading in the dataset, we used the R package readxl. For the creation of the plots, we used ggplot2 and ggpubr. Antithrombin III activity was analyzed for 2 separate groupings of patients. The first grouping separated the patients based on whether they had a DVT or not and the second separated the patients based on whether they had a hemorrhage or not. Based on the fact that the distribution of antithrombin III activity was nonnormal, Wilcoxon rank sum tests were carried out for these 2 groupings. These tests compared the median values of the DVT group vs the no DVT group based on antithrombin III activity; the median values of hemorrhage group against the no hemorrhage group were also based on antithrombin III activity. Furthermore, Wilcoxon rank sum tests for the difference in median antithrombin III activity at time points were carried out for both sets. Finally, for both sets, Wilcoxon rank sum tests were carried out comparing the median values of the time points and the group membership for each set.

Statistical analyses were carried out for comparison of groups deemed deficient in antithrombin III (defined as <80% activity) vs those nondeficient in antithrombin III activity (≥80% activity) based on observed patient variables (eg, age, weight, and mechanism of injury). Where the dependent variable was quantitative and normally distributed, a *t* test was used. Where the dependent variable was quantitative and not normally distributed, a Wilcoxon rank sum test of median values was used. For categorical variables, a χ^2^ test was used when the expected values were not too low and where the contingency tables were greater than 2 × 2. For 2 × 2 contingency tables, a Fisher exact test was used. *P* values were reported as calculated without correction but were compared with a global type I error rate of 0.0006. This rate was arrived at by using the Bonferroni-correction method. The threshold for statistical significance was determined by considering that there were 81 hypotheses conducted on the dataset. Thus, by the Bonferroni method, α = (.05/81) = .0006. All of the tests were 2-sided.

## Results

The cohort for this study included 292 patients (mean [SD] age, 54.4 [19.0] years; 211 men [72.2%] and 81 women [27.7%]) (Table 1). Most patients (170 of 292 [58.2%]) received enoxaparin. There was no difference in age, sex, body mass index, systolic blood pressure, or mechanism of injury at the time of admission between patients who were antithrombin III deficient at any point during the first 6 days of the hospitalization and those who were not. Most patients (273 of 292 [93.5%]) had blunt injuries. At admission, 14.0% of patients (41 of 292) were antithrombin III deficient, and 42.5% of patients (124 of 292) were deficient at some time point during the first 6 days of admission. Antithrombin III deficiency was associated with a lower mean (SD) admission Glasgow Coma Scale score (12.6 [3.8] vs 14.0 [2.4]; *P* = 1.10 × 10^−19^).

**Table 1.** Patient Baseline Characteristics Between Study Groups

Characteristic	Not antithrombin III deficient (n = 159)	Antithrombin III deficient (n = 133)	*P* value (test)^a^
Age, mean (SD), y	54.6 (18.6)	53.8 (19.5)	.75 (Wilcoxon)
Sex, No (%)			
Male	111 (69.8)	100 (75.2)	.36 (Fisher exact)
Female	48 (30.2)	33 (24.8)	
Weight, mean (SD), kg	83.0 (20.0)	85.9 (21.5)	.24 (Wilcoxon)
BMI, mean (SD)	27.4 (6.1)	29.1 (10.1)	.07 (Wilcoxon)
SBP, mean (SD), mm Hg	137.8 (24.7)	138.9 (30.4)	.45 (Wilcoxon)
Prehospital GCS score, mean (SD)	14.0 (2.4)	12.6 (3.8)	1.10 × 10^−19^ (Wilcoxon)
ISS, mean (SD)	14.8 (7.5)	18.9 (10.9)	.002 (Wilcoxon)
ISS group, No. (%)			
0-14	97 (61.0)	59 (44.4)	.001 (χ^2^)
15-25	45 (28.3)	40 (30.1)	
>25	17 (10.7)	34 (25.6)	
Mechanism of injury, No. (%)			
Blunt	151 (95.0)	122 (91.7)	.34 (Fisher exact)
Penetrating	8 (5.0)	11 (8.3)	
Prehospital fluids, No. (%)	43 (27.0)	60 (45.1)	.001 (Fisher exact)
Trauma level, No. (%)			
Level I	16 (10.1)	36 (27.1)	.0003 (χ^2^)
Level II	33 (20.8)	30 (22.6)	
Level III	110 (69.2)	67 (50.4)	
AIS score, head or neck, No. (%)			
0	72 (45.3)	55 (41.3)	.41 (χ^2^)
1	4 (2.5)	1 (0.8)	
2	28 (17.6)	19 (14.3)	
3	29 (18.2)	25 (18.8)	
4	17 (10.7)	19 (14.3)	
5	9 (5.7)	14 (10.5)	
AIS score, face, No. (%)			
0	135 (84.9)	99 (74.4)	.05 (χ^2^)
1	13 (8.2)	12 (9.0)	
2	10 (6.3)	19 (14.3)	
3	1 (0.6)	3 (2.3)	
AIS score, chest, No. (%)			
0	73 (45.9)	62 (46.6)	.20 (χ^2^)
1	6 (3.8)	2 (1.5)	
2	12 (7.5)	4 (3.0)	
3	58 (36.5)	50 (37.6)	
4	9 (5.7)	11 (8.3)	
5	1 (0.6)	4 (3.0)	
AIS score, abdomen or pelvis, No. (%)			
0	125 (78.6)	88 (66.2)	.17 (χ^2^)
1	2 (1.3)	1 (0.8)	
2	20 (12.6)	28 (21.0)	
3	8 (5.0)	12 (9.0)	
4	4 (2.5)	3 (2.3)	
5	0	1 (0.8)	
AIS score, extremities, No. (%)			
0	90 (56.6)	64 (48.1)	.46 (χ^2^)
1	3 (1.9)	3 (2.3)	
2	47 (29.5)	42 (31.6)	
3	17 (10.7)	20 (15.0)	
4	2 (1.2)	2 (1.5)	
5	0	2 (1.5)	

Abbreviations: AIS, abbreviated injury scale; BMI, body mass index (calculated as weight in kilograms divided by height in meters squared); GCS, Glasgow Coma Scale; ISS, Injury Severity Score; SBP, systolic blood pressure.

^a^ Statistically significant after Bonferroni-correction test.

Patients with antithrombin III deficiency were significantly more likely to have received a transfusion with fresh frozen plasma and/or red blood cells before the first 8 hours (eTable 1 in Supplement 1). Antithrombin III deficiency was associated with elevated fibrinogen levels (antithrombin III deficiency, 64.7% [86 of 133]; no antithrombin III deficiency, 42.8% [68 of 159]; *P* = .0003) (eTable 2 in Supplement 1). There was also a significant association between antithrombin III deficiency and thrombocytopenia (antithrombin III deficiency, 24.8% [33 of 133]; no antithrombin III deficiency, 5.0% [8 of 159]; *P* = 1.94 × 10^−6^).

We investigated antithrombin III deficiency up to the first 6 days of hospitalization to capture a sufficient number of patients to analyze prior to hospital discharge. Antithrombin III deficiency at any point during the first 6 days of hospitalization was associated with fewer mean (SD) ventilator-free days (27.8 [5.1] vs 29.6 [1.4] days; *P* = .0003), hospital-free days (20.3 [8.2] vs 24.0 [5.7] days; *P* = 1.37 × 10^−6^), and ICU-free days (25.7 [4.9] vs 27.7 [2.3] days; *P* = 9.38 × 10^−6^) (Table 2; eTable 3 in Supplement 1). There was no correlation of the timing of antithrombin III deficiency during the first 6 days with ventilator time, hospital time, or ICU time.

**Table 2.** Complications in Trauma Patients

Variable	All trauma patients			Deficient patients			Test
	Antithrombin III deficient (n = 133)	Not antithrombin III deficient (n = 159)	*P* value	At baseline	Later in timeline	*P* value	
Sex, No./total No. (%)							
Male	100/133 (75.2)	111/159 (69.8)	.36 (Fisher exact)	30 (30.0)	70 (70.0)	.83	Fisher exact
Female	33/133 (24.8)	48/159 (30.2)		11 (33.3)	22 (66.7)		
Ventilator-free, mean (SD), d	27.8 (5.1)	29.6 (1.4)	.0003^a^	27.8 (4.4)	27.8 (5.6)	.81	Wilcoxon
Hospital-free, mean (SD), d	20.3 (8.2)	24.0 (5.7)	1.37 × 10^−6^^a^	19.8 (11.3)	20 (8.5)	.16	Wilcoxon
ICU-free, mean (SD), d	25.7 (4.9)	27.7 (2.3)	9.38 × 10^−6^^a^	24.9 (6.1)	26.0 (4.2)	.88	Wilcoxon
ICH, No./total No. (%)	61/133 (45.9)	71/159 (44.7)	.93	19/61 (31.1)	42/61 (68.9)	.05	Fisher exact
Neurosurgical intervention, No./total No. (%)	14/61 (22.9)	15/159 (9.4)	.90	5/14 (35.7)	9/14 (64.3)	.70	Fisher exact
Progressive ICH, No./total No. (%)	28/133 (21.0)	10/159 (6.3)	.0003^a^	9/28 (32.1)	19/28 (67.9)	.28	Fisher exact
Other hemorrhage, No./total No. (%)	22/133 (16.5)	9/159 (5.7)	.005	8/22 (36.4)	14/22 (63.6)	.54	Fisher exact
DVT, No./total No. (%)	16/133 (12.0)	10/159 (6.3)	.47	5/16 (31.3)	11/16 (68.7)	.47	Fisher exact
PE, No./total No. (%)	3/133 (2.2)	1/159 (0.6)	.49	1/3 (33.3)	2/3 (66.7)	>.99	Fisher exact
DVT to PE, No./total No. (%)	3/16 (19.0)	1/10 (10.0)	.99	1/3 (33.3)	2/3 (66.7)	>.99	Fisher exact
Mortality, No./total No. (%)	12/133 (9.0)	2/159 (1.3)	.004	5/12 (41.7)	7/12 (58.3)	.004	Fisher exact

Abbreviations: DVT, deep vein thrombosis; ICH, intracranial hemorrhage; ICU, intensive care unit; PE, pulmonary embolism.

^a^ Statistically significant after Bonferroni-correction test.

There was no association of antithrombin III deficiency with ICH or the need for neurosurgical intervention (Table 2). However, antithrombin III deficiency was associated with progression of ICH (21.0% [28 of 133] vs 6.3% [10 of 159]; *P* = .0003). The timing of antithrombin III deficiency was not associated with ICH incidence, progression, or neurosurgical intervention. Antithrombin III deficiency showed a nonsignificant association with mortality (12 of 133 [9.0%] vs 2 of 159 [1.3%]; *P* = .004) (Table 2) but did not reach significance using a Bonferroni-corrected type I error rate of 0.0006. Most deaths (11 of 14) resulted from catastrophic brain injury, including progression of ICH as well as 1 incidence of anoxic brain injury without ICH.

**Figure 3.**
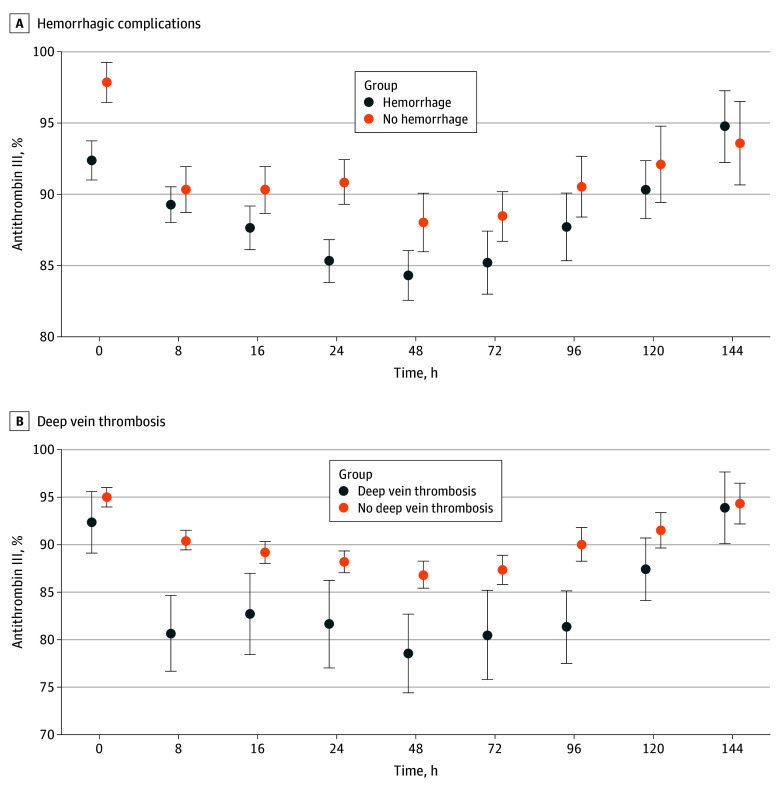
Thrombohemorrhagic Complications With Respect to Antithrombin III Deficiency Over Time

There was no higher rate of DVT in the antithrombin III–deficient group (Table 2) in univariable analysis. There was also no higher rate of PE in the antithrombin III–deficient group, and antithrombin III deficiency was not associated with progression of DVT to PE. We therefore investigated whether antithrombin III levels differed significantly at any time during hospitalization. Patients with DVT did not have significantly different antithrombin III levels on admission but showed a precipitous decrease in antithrombin III levels at 8 hours that remained significantly lower at 16, 24, 48, 72, and 96 hours after admission (Figure 3B).

## Discussion

Trauma patients exhibit a complex and incompletely understood balance of coagulopathy that can lead to both devastating bleeding and thrombotic complications. Trauma-induced coagulopathy is a result of a combination of traumatic injury, hemorrhage, immediate activation of hemostatic mechanisms, and consumption of factors with increased fibrinolysis that is exacerbated by hypothermia, acidosis, transfusion, and dilution with crystalloid.^18^ This acute physiological derangement may be associated with hemorrhage-related deaths among trauma patients within the first 24 hours after injury. Ultimately, a transition is thought to occur after hemorrhage control that is characterized by the escape of localized thrombin activation and systemic inflammation, making these same patients hypercoagulable, with an incidence of VTEs as high as 58%.^5,19,20^ These changes indicate the importance of further understanding the potential markers and factors that can help physicians navigate and anticipate therapeutic strategies.

Acquired deficiency of antithrombin III has emerged as a pathophysiologic derangement of interest in trauma-induced coagulopathy. Antithrombin III is a primary circulating anticoagulant that undergoes 1:1 binding with the exponentially increasing amounts of thrombin (among other serine protease clotting factors) in trauma before being cleared from the circulation. Low-molecular-weight heparin thromboprophylaxis, recommended by the American College of Chest Physicians and Eastern Association for the Surgery of Trauma, acts by potentiating the natural anticoagulant activity of antithrombin III.^4,21,22,23^ Despite its use, studies have suggested that approximately 30% of patients receiving appropriate thromboprophylaxis still experience VTE-related complications during their hospitalization^24^ and that approximately 80% of trauma patients who have had a VTE-related complication are receiving appropriate thromboprophylaxis.^13^ Furthermore, antithrombin III is emerging as an important regulator of vascular barrier function,^11,14^ highlighting its potential importance in trauma-related thrombohemorrhagic complications.

Antithrombin III has been shown to be important for protection of endothelial syndecan expression and barrier function.^11^ Among patients with trauma in Japan, it has been shown that early antithrombin III deficiency in trauma is associated with vascular leak.^14^ The same study also showed that this adverse effect was not associated with antithrombin III consumption in thrombin-antithrombin complexes but rather with inflammation-regulated endothelial damage and albumin depletion. In our study, patients with antithrombin III deficiency had a significantly lower prehospital Glasgow Coma Scale score than those with no deficiency (12.6 vs 14.0; *P* = 1.10 × 10^−19^). In our study, hemorrhagic complications were not associated with prolongation of the prothrombin time, activated partial thromboplastin time, or fibrinogen levels below the critical threshold,^25^ arguing against a consumptive or dilutional component in this population. These findings may be reflective of trauma-related endotheliopathy and the role of antithrombin III in barrier function.^11,14,26^ However, it could also mean that antithrombin III deficiency occurs among more severely injured patients with endotheliopathy of trauma.

### Limitations

This study has some limitations. It excluded one-third of the enrolled participants because of declined consent or hospital discharge prior to obtaining consent, limiting the size of the studied cohort, which could have affected the results and conclusions. Many patients did not contribute samples at every time point due to rapid recovery and earlier discharge than others in the cohort. Similarly, some older patients and/or patients with severe injuries opted to transition to comfort care, halting data and sample collection, or expressed wishes not to undergo invasive procedures proposed by the treating teams. This loss of data points and deviation from the standard of care could bias the results.

As with many other studies examining antithrombin III levels in trauma and its associations with outcomes, our study conclusions are limited by the many variables associated with antithrombin III levels and activity. Aspects of trauma patient condition and management, including hemorrhage, trauma-induced consumptive coagulopathy, cleavage and inactivation by neutrophil elastase, autoheparinization secondary to glycocalyx shedding, endotheliopathy, volume resuscitation, thromboprophylaxis, blood product transfusion, and other variables, are associated with antithrombin III levels.^27,28,29^ Nonetheless, the frequency of antithrombin III deficiency in our trauma patient cohort fell within previously reported prevalence rates of 30% to 62% among trauma patients,^13,27,30^ and we also found that the highest rates of incidence of antithrombin III deficiency occurred at time points within the first 24 hours, consistent with the prevalence of the mechanisms listed, which are most prevalent during that timeline.

Comparisons between trauma studies can be limited by the type of trauma center, patient population, ability to obtain consent, thromboprophylaxis protocols, and the use of screening, as well as other variables. Our inclusion criteria limited enrollment to highly injured trauma patients and showed that antithrombin III–deficient patients overall were more severely ill, with lower prehospital Glasgow Coma Scale scores, fewer ventilator-free days, fewer hospital-free days, and fewer ICU-free days compared with the group with normal antithrombin III levels. The mean Injury Severity Scores of our cohort were most closely matched with the 1994 study by Miller and colleagues,^13^ which found similar correlations. A correlation between Injury Severity Scores and antithrombin III deficiency was not found in a subsequent 1996 study by Owings et al,^27^ which noted an increase in antithrombin III levels over time in their cohort with brain injury. Although it is difficult to fully explain these observations, it is possible that the alternative thromboprophylaxis approaches may have played a role, as heparin-based thromboprophylaxis has been shown to have the capacity to affect antithrombin III levels.^28^ In our study, most patients (58.2%) received enoxaparin, compared with the infrequent use of chemoprophylaxis (3%) in the study by Owings et al^27^ that primarily used mechanical thromboprophylaxis (83%). Furthermore, most patients in our study population (94.0%) had blunt injuries, which may have been different in the other study.

Trauma-induced coagulopathy studies are further complicated by the fact that both coagulation and inflammation are intertwined processes in trauma, as is antithrombin III function in these processes and possible outcomes.^31,32,33^ Specifically, antithrombin III has been shown to regulate human neutrophil migration and thus decrease effects of oxidative burst.^34^ Furthermore, antithrombin III infusion in animal models can protect against ischemia or reperfusion injury^35,36^ and, in ex vivo human studies involving trauma patients, can potentiate protective effects of enoxaparin.^37^ Upregulation of the acute inflammatory reactant fibrinogen is a known phenomenon that can affect the effectiveness of thromboprophylaxis^38^ as well as contribute to vascular dysfunction and other complications in traumatic brain injury.^39^ In our study, we also found a strong correlation between fibrinogen elevation and antithrombin III deficiency as well as between fibrinogen elevation and most DVTs, and over half of hemorrhagic complications. Recent studies showed that low antithrombin III levels were inversely proportional to incidence of VTE, length of hospital stay, and mortality,^40^ which is confirmed and extended by our results. Further mechanistic studies, however, would need to be performed to delineate a cause and effect of this association as well as others.

This study evaluated a broad population of trauma patients that included patients with both blunt and penetrating mechanisms. Most of the patients evaluated in this study (94.0%) had a blunt mechanism, so we do not believe that patients with a penetrating mechanism present a substantial bias in these data.

## Conclusions

In this cohort study of trauma patients, antithrombin III deficiency was associated with greater severity of illness and worse hospital outcomes. Specifically, antithrombin III deficiency was associated with fewer ventilator-free days, hospital-free days, and ICU-free days. Antithrombin III–deficient patients had a significantly higher incidence of progressive hemorrhage, highlighting a potential differential role for antithrombin III in trauma-related early endotheliopathy. Although antithrombin III deficiency was not significantly associated with DVT, patients who developed a DVT had a more precipitous decrease in antithrombin III levels that were significantly lower than patients who did not develop a DVT. These findings suggest that monitoring of antithrombin III levels may be warranted for trauma patients.

## Notes

Supplement 1. **eFigure.** Inclusion and Exclusion Criteria **eTable 1.** Number of Subjects That Received a Transfusion **eTable 2.** Fibrinogen and Platelet Levels in Trauma Patients **eTable 3.** Outcomes of DVT/PE, Hemorrhage and Mortality

Supplement 2.
Data Sharing Statement
