# Supplementary material for: Antithrombin III Levels and Outcomes Among Patients With Trauma
Source: JAMA Netw Open. 2024 Aug 15;7(8):e2427786. doi: 10.1001/jamanetworkopen.2024.27786 (PMC11327888; doi:10.1001/jamanetworkopen.2024.27786)
Supplement: Supplement 1. — eFigure. Inclusion and Exclusion Criteria eTable 1. Number of Subjects That Received a Transfusion eTable 2. Fibrinogen and Platelet Levels in Trauma Patients eTable 3. Outcomes of DVT/PE, Hemorrhage and Mortality [file jamanetwopen-e2427786-s001.pdf]

Supplementary Online Content

Farrell DH, McConnell KM, Zilberman-Rudenko J, et al. Antithrombin III levels and outcomes among patients with trauma. *JAMA Netw Open.* 2024;7(8):e2427786. doi:10.1001/jamanetworkopen.2024.27786

eFigure. Inclusion and Exclusion Criteria
eTable 1. Number of Subjects That Received a Transfusion
eTable 2. Fibrinogen and Platelet Levels in Trauma Patients
eTable 3. Outcomes of DVT/PE, Hemorrhage and Mortality

This supplementary material has been provided by the authors to give readers additional information about their work.

© 2024 Farrell DH et al. *JAMA Network Open.*

eFigure. Inclusion and Exclusion Criteria

Inclusion Criteria

Any trauma activation meeting one of the following:

- GCS <12 or ICH confirmed on initial CT.
- Systolic blood pressure <90 mmHg.
- Any penetrating injury to the neck, torso or groin.
- Amputation proximal to the ankle or wrist.
- Uncontrolled external hemorrhage.
- ≥2 long bone fractures.
- Pelvic fracture.
- Paraplegia or quadriplegia from injury at admit.
- Combination trauma with burns <20% TBSA.
- ≥3 rib fractures.
- Any solid organ injury.

Exclusion Criteria

If meeting any of the following criteria:

- Discharged from the Emergency Department.
- Age <18 years.
- Invasive procedure prior to arrival at OHSU.
- Transfusion of blood products prior to arrival at OHSU.
- Failure to have initial samples drawn in first hour.
- Enrolled in a prehospital interventional study.
- Prisoners – defined as anyone directly admitted from a correctional facility.

© 2024 Farrell DH et al. *JAMA Network Open.*

eTable 1. Number of Subjects That Received a Transfusion

FFP

Not deficient			Deficient		
	n	(%)	n	(%)	*p-value*
On arrival	0	0	6	4.5	0.02
8 Hrs	0	0	0	0	1
16 Hrs	0	0	0	0	1
24 Hrs	0	0	0	0	1
48 Hrs	0	0	0	0	1
3 days	0	0	0	0	1
4 days	0	0	1	0.7	0.91
5 days	0	0	0	0	1
6 days	0	0	1	0.7	0.91

Platelets

Not deficient			Deficient		
	n	(%)	n	(%)	*p-value*
On arrival	1	0.6	3	2.2	0.49
8 Hrs	0	0	0	0	1
16 Hrs	0	0	0	0	1
24 Hrs	0	0	0	0	1
48 Hrs	0	0	0	0	1
3 days	0	0	0	0	1
4 days	0	0	1	0.7	0.91
5 days	0	0	0	0	1
6 days	0	0	1	0.7	0.91

RBCs

Not deficient			Deficient		
	n	(%)	n	(%)	*p-value*
On arrival	0	0	9	6.7	0.001
8 Hrs	0	0	0	0	1
16 Hrs	0	0	0	0	1
24 Hrs	0	0	0	0	1
48 Hrs	0	0	0	0	1
3 days	1	0.6	6	4.5	0.07
4 days	1	0.6	8	6.0	0.02
5 days	1	0.6	3	2.2	0.49
6 days	2	1.2	5	2.7	0.31

© 2024 Farrell DH et al. *JAMA Network Open.*

eTable 2. Fibrinogen and Platelet Levels in Trauma Patients

Not Deficient			Deficient			Significant
Male, No (%)	111	69.8	100	75.2	0.36 (Fisher's Exact)	
Female, No (%)	48	30.2	33	24.8		
**Fibrinogen**	n	(%)	n	(%)	*p-value*	
< 100 mg/dL	2	1.2	6	4.5	0.18	
> 500 mg/dL	68	42.8	86	64.7	.00028	X
**Platelets**	n	(%)	n	(%)	*p-value*	
$< 100 \times 10^9$/L	8	5.0	33	24.8	1.94×10^{-6}	X
$> 450 \times 10^9$/L	2	1.2	3	2.2	0.83	

© 2024 Farrell DH et al. *JAMA Network Open.*

eTable 3. Outcomes of DVT/PE, Hemorrhage and Mortality

	**DVT/PE**	**Neither**		**Hemorrhage**	**No Hemorrhage**		**Mortality**	**No Mortality**	
	Mean ± sd	Mean ± sd	*p-value*	Mean ± sd	Mean ± sd	*p-value*	Mean ± sd	Mean ± sd	*p-value*
Vent free days	25.2 ± 5.9	29.1± 3.3	5.32×10^{-7}	28.2 ± 4.7	29.6 ± 1.4	0.003	23.9 ± 3.9	29.0 ± 3.5	2.02×10^{-11}
Hosp free days	12.5 ± 10.2	23.2± 6.1	1.97×10^{-7}	21.3 ± 8.2	23.5 ± 5.3	0.27	23.8 ± 3.9	22.3 ± 7.2	0.98
ICU free days	22.9 ± 5.1	27.2 ± 3.5	5.73×10^{-7}	25.9 ± 4.6	27.9 ± 2.2	3.49×10^{-8}	23.8 ± 3.8	27.0 ± 3.8	0.002
ATIII Activity	83.7 ± 17.2	90.5 ± 17	4.00×10^{-7}	88.4 ±17.7	91.6 ±16.2	0.0008	73.9 ± 17.4	90.7 ±16.6	1.62×10^{-16}

Wilcoxon tests carried out on all tests of distributions since the data tested far from normality according to the Shapiro test.

© 2024 Farrell DH et al. *JAMA Network Open.*
