# Supplementary material for: Antithrombin III Levels and Outcomes Among Patients With Trauma
Source: JAMA Netw Open. 2024 Aug 15;7(8):e2427786. doi: 10.1001/jamanetworkopen.2024.27786 (PMC11327888; doi:10.1001/jamanetworkopen.2024.27786)
Supplement: Supplement 2. — Data Sharing Statement [file jamanetwopen-e2427786-s002.pdf]

Data Sharing Statement

Farrell. Antithrombin III Levels and Outcomes Among Patients With Trauma. *JAMA Netw Open*. Published August 15, 2024. doi:10.1001/jamanetworkopen.2024.27786

Data
Data available: Yes
Data types: Deidentified participant data, Data dictionary
How to access data: schreibm@ohsu.edu
When available: With publication

Supporting Documents
Document types: None

Additional Information
Who can access the data: researchers whose proposed use of the data has been approved
Types of analyses: for any purpose
Mechanisms of data availability: signed data access agreement
